# A Rose Extract Protects the Skin against Stress Mediators: A Potential Role of Olfactory Receptors

**DOI:** 10.3390/molecules25204743

**Published:** 2020-10-16

**Authors:** Romain Duroux, Anne Mandeau, Gaelle Guiraudie-Capraz, Yannick Quesnel, Estelle Loing

**Affiliations:** 1 Department of Research and Development, International Flavors and Fragrances-Lucas Meyer Cosmetics, 31036 Toulouse CEDEX, France; anne.mandeau@lucasmeyercosmetics.com; 2 Institute of Neurophysiopathology, CNRS, Aix-Marseille University, UMR 7051, CEDEX 15, F-13344 Marseille, France; gaelle.guiraudie@univ-amu.fr; 3 ChemCom S.A., B-1070 Brussels, Belgium; yaq@chemcom.be; 4 Department of Research and Development, International Flavors and Fragrances-Lucas Meyer Cosmetics, Quebec, QC G1V 4M6, Canada; estelle.loing@iff.com

**Keywords:** olfactory receptors, rose extract, stress, cAMP, keratinocytes, skin explants

## Abstract

Olfactory receptors (ORs) are expressed and active in various human tissues, including the skin. Although the sense of smell plays an important physiological role in the regulation of mood and stress, a link between olfactive compounds, ORs, and skin stress has yet to be established. This study aims to investigate the role of newly identified skin ORs and agonists in the modulation of skin stress. Screening for odorant molecules was done with cAMP functional assay to identify OR agonists. RT-qPCR and immunofluorescence microscopy were conducted to identify and quantify ORs in epidermal keratinocytes (NHEKs) and human skin explants, as well as to evaluate specific markers (G6PDH, loricrin, and γH2AX) of stress-induced skin alterations. A randomized double-blinded, split-face clinical study was performed on a panel of stressed women to measure the benefits of OR agonist treatment for skin. Three new ORs (OR10A6, OR2AG2, and OR11H4) were identified in skin. A specific Rose extract and its major constituent (phenylethyl alcohol) were found to activate these ORs. The extract composition was revealed by both GC/FID and GC/MS analyses simultaneously and showed the presence of 34 volatiles molecules. Moreover, epinephrine induces a skin stress response characterized by increased expression of G6PD, loricrin, and γH2AX biomarkers, and a decrease of OR expression. These effects were prevented in the presence of rose extract and its benefits were confirmed clinically by a decrease in the appearance of under-eye dark circles. Altogether, our findings suggest that ORs may represent a new, promising way to treat stress-associated skin disorders.

## 1. Introduction

Olfactory receptors (ORs), discovered in 1991 by Buck and Axel [1], belong to the G-protein coupled receptor family (GPCR). ORs are encoded by the largest multigene family in mammals with 396 intact OR genes and 425 OR pseudogenes corresponding to a sequence with a nonsense mutation, frameshift, and deletion within conserved regions [2]. ORs are mainly located in the olfactory epithelium and mediate the first step in odor recognition [3,4,5]. Several studies suggest that every human individual is characterized by a different combination of OR-segregating pseudogenes [2]. This genetic variation in the coding regions of human OR genes contributes to the variation in odor perception among individuals [6,7]. Since their original discovery in the nasal cavity, ORs have been found in various other cell types throughout the body where they might regulate physiological cell functions beyond olfaction [8,9]. Indeed, an increasing number of studies have uncovered ectopic expression of ORs in a variety of organs, including prostate, tongue, heart, testis, brain, gut, hair, and skin [10,11,12,13,14,15,16]. Nevertheless, the physiological roles of these new non-nasal ORs remains to be elucidated. For this purpose, their ligands must first be identified, since numerous ectopic ORs are still orphan receptors.

Benefits of odorant molecules for the skin are now well recognized. Indeed, the expression of at least five different OR transcripts (OR2AT4, OR6M1, OR5V1, OR11A1, and OR6V1) has been described in human epidermis [16]. Among these receptors, activation of OR2AT4 by Sandalore^®^ (a synthetic sandalwood odorant) was demonstrated to promote keratinocyte migration and proliferation, as well as wound re-epithelialization. For its part, OR51B5 seems to promote wound healing by supporting both keratinocyte migration and inflammatory processes, with secretion of cytokines, when stimulated with agonists such as the isononyl alcohol or the cyclohexyl salicylate [17]. Moreover, melanocytes present in the basal layer of the skin epidermis express OR51E2 [18] and OR2A4/7 [19]. A role in the biosynthesis of melanin is suspected for these two receptors through increased levels of intracellular cAMP and Ca^2+^ following their activation by odorant ligands [19]. Being directly exposed to the environment, the skin is highly susceptible to environmental stressors. Chronic stress exposure leads to skin exhaustion through persistent epinephrine release [20,21]. In the long term, epinephrine damages the skin and precipitates ageing, mostly via skin barrier disruption and increased DNA-damage, following activation of β2 adrenergic receptors (ADRB2) [22,23]. In aromatherapy, essential oils and odorants are well-known for their relaxing and anti-stress effects in humans, following inhalation or transdermal absorption [24]. Inhalation of odorants has been described as influencing skin functions, including cutaneous barrier permeability and sebum secretion [25]. However, the role of ORs in this response is rarely mentioned. The aim of the present study is to address the question of a potential involvement of skin ORs in the handling of chronic skin stress. 

Our results identify three novel ORs expressed in human skin, namely, OR10A6, OR11H4, and OR2AG2. Moreover, we found that a rose extract and its main component, phenylethyl alcohol, act as agonists for these three ORs. We also provide evidence that these OR activators protect skin against stress both ex vivo and in vivo. Taken together, our work suggests that skin olfactory receptors are involved in stress response mechanisms and that the tested rose extract efficiently protects the skin against stressors. 

## 2. Results

### 2.1. Chemical Composition of Rose Extract

The chemical structures of the compounds (Table 1) were elucidated by GC/MS and GC/FID, resulted in the identification of 34 components representing 96.86% of the total extract. The remaining percentage corresponds to unknown molecules. The analysis revealed that the rose extract contains 56.7% of phenylethyl alcohol as a major molecule. In detail, four other main peaks of the extract were recorded corresponding to citronellol (16.7%), geraniol (8.90%), nerol (4.6%), and nonadecane (3.8%). Many other terpenes were also observed at a trace level (Table 1).

### 2.2. ORs are Expressed on Human Keratinocytes and Skin Explants

**Figure 1:**
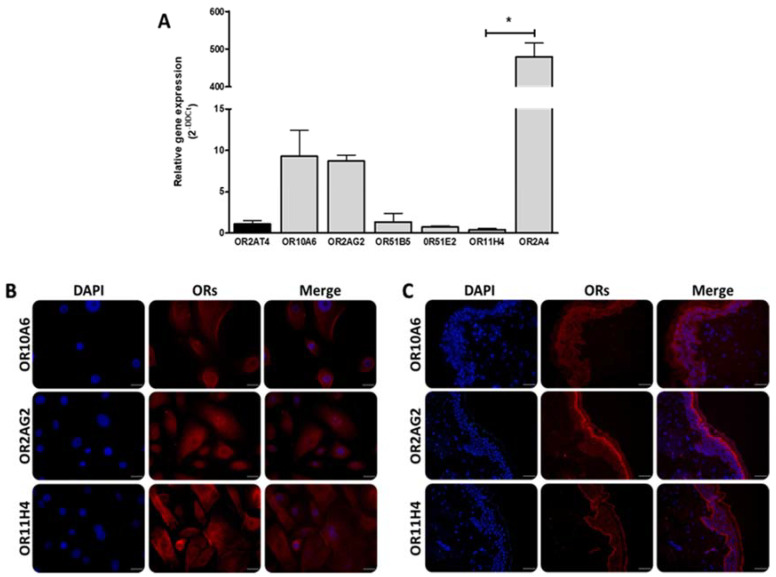
Olfactory receptor expression in NHEK cells. (**A**) mRNA expression of olfactory receptors was measured using RT-qPCR (*n* = 3). Data are presented as mean ±SEM. Significance was calculated using the Kruskall–Wallis test (* *p* ≤ 0.05). (**B**) Immunodetection of olfactory receptors in human keratinocytes. Scale bar: 15 µm. (**C**) Immunohistochemistry of olfactory receptors in human skin explants. Scale bar: 50 µm (for a scale bar: 15 µm, see Supplementary Materials Figure S1). The distribution of ORs expression was observed with a Zeiss microscope. Red: ORs, Blue: nucleus.

To investigate the general expression of ORs in cultured human primary keratinocytes, we analyzed the presence at the RNA level of randomly selected ORs using quantitative reverse transcription-polymerase chain reaction (RT-qPCR) (Figure 1A). Seven out of the 16 ORs selected are found to be expressed in NHEK cells. OR1D2, OR2J3, OR2J2, OR1A1, OR4D1, OR1G1, OR10J5, OR2W1, and OR10G4 are not found whereas the expression of known skin ORs (OR51B5, OR51E2, OR2A4, and OR2AT4) is confirmed with a higher expression of OR2A4 compared to OR2AT4. Interestingly, we observed mRNA expression of three ORs (OR10A6, OR11H4, and OR2AG2) never previously described in skin. OR10A6 and OR2AG2 expression is predominant over OR2AT4 whereas OR11H4 shows a lower expression. Fold change values were 9.3 ± 1.9 (OR10A6), 8.7 ± 0.8 (OR2AG2) and 0.4 ± 0.28 (OR11H4). 

Immunofluorescence microscopy analysis was performed on primary human keratinocytes and human skin explants to confirm the presence of these 3 receptors on skin (Figure 1B,C). Interestingly, in normal skin sections, different expression levels and different sub-localization are observed for each of the three olfactory receptors (ORs). While OR11H4 staining is mainly restricted to the basal membrane of the epidermis, OR2AG2 is rather expressed in the suprabasal layer, and OR10A6 is found throughout the whole epidermis with more intensive detection in the suprabasal layer. 

### 2.3. Rose Extract and Phenylethyl Alcohol Activate Skin Human Olfactory Receptors

Measurement of intracellular levels of cAMP on HEK293T-RTP1S/RTP2 cells transfected with either a plasmid encoding the indicated odorant receptor (OR10A6, OR2AG2, or OR11H4), or an empty vector, was used to detect olfactory receptor activity (see Supplementary Materials Figure S2). A total of 18 compounds were screened. Dose-response curves were constructed for selected hits and the EC50 values were determined. These compounds were randomly selected based on their naturality, their capacity to bind ORs based on data from the literature, and on the likelihood of finding these molecules with a high percentage in flowers extracts.

All scenarios were obtained (Table 2). Four compounds failed to activate the overexpressed ORs. This was the case for Benzyl alcohol (Table 2 entry 4) and Lavandulol (Table 2 entry 15). Cis-3-hexenol (Table 2, entry 3) was the only compound to solely activate OR2AG2, whereas Cyclamen aldehyde (Table 2, entry 8), Lyral (Table 2, entry 9), Nonadecane (Table 2, entry 18), and α-Ionone (Table 2, entry 11) specifically activated OR10A6. Interestingly, an isomer of the latter (β-Ionone) which presents a different odor, was not found to be an OR10A6 agonist (Table 2, entry 14). Seven compounds activated two skin ORs, and systematically the same two (OR10A6 and OR2AG2). To our knowledge, these two receptors are responsive to a large number of compounds, not necessarily structurally linked, and can be considered as broadly tuned “generalist” ORs. On the contrary, OR11H4 is more selective, with only Phenylethyl alcohol (PEA, Table 2, entry 12) and Phenyl propyl alcohol (PEA, Table 2, entry 13) as activators, with EC_50_ values of 20.3 µM and 52.84 µM, respectively. The fact that Benzyl alcohol (Table 2, entry 4) does not activate OR11H4 demonstrates the importance of having at least two carbons between the phenyl ring and the alcohol function. 

PEA is most abundant in rose flower. For this reason, a rose extract containing around 56% of PEA was chosen to test OR activation. As expected, the in vitro functional assay revealed that this rose extract is able to induce a transient increase in cAMP in HEK293T cells transfected with OR11H4, OR2AG2 and OR10A6, with EC_50_ values of 1.65 × 10^−4^%, 8.12 × 10^−4^% and 1.51 × 10^−4^% respectively (Table 2, entry 19).

**Figure 2:**
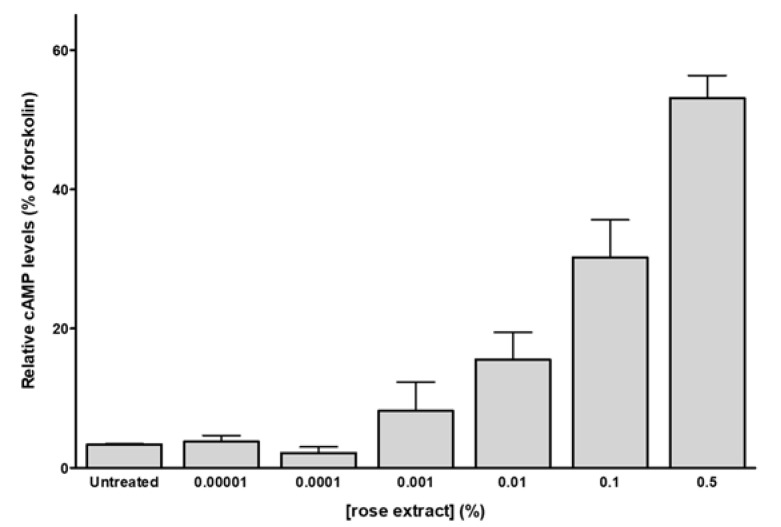
Rose extract dose-dependently increases intracellular cAMP in NHEK cells after 30 min of stimulation. Data are expressed as mean ±SEM values of percent cAMP activity over forskolin (positive control, 10 µM, (*n* = 3)).

Previous OR studies in keratinocytes have demonstrated that OR agonists can activate the cAMP intracellular pathway [18,19]. Accordingly, within 30 min of application, the rose extract increased cAMP levels in NHEK (Figure 2). The dose-response curve shows that rising rose extract concentrations (1 × 10^−5^ to 0.5%) correlate with an increase in intracellular cAMP levels, up to 53 ± 8% relative to forskolin (10 µM) serving as positive control. 

### 2.4. OR Agonists Restore Olfactory Receptor Expression Following Downregulation with Epinephrine and Protects the Skin against Stress

**Figure 3:**
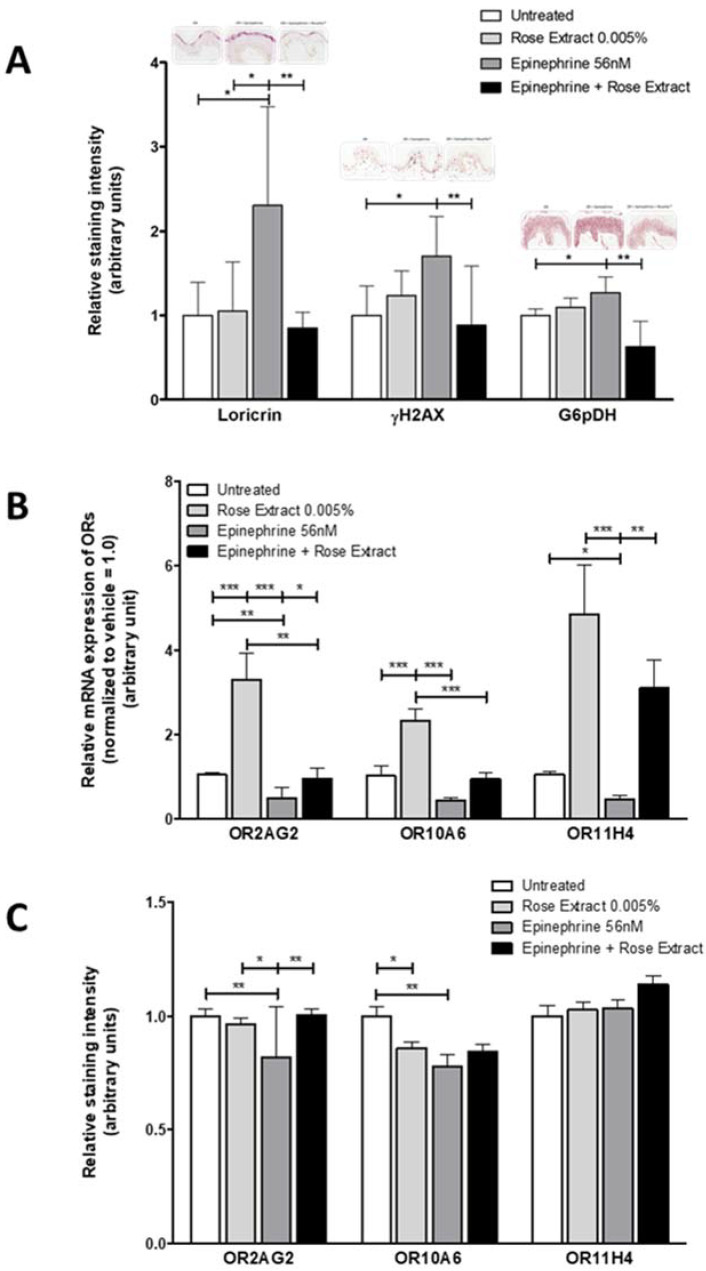
Effect of rose extract (5 × 10^−3^%) on epinephrine–induced (56 nM) stress markers and OR expression in skin explants. (**A**) Epinephrine-induced stress for 9 days significantly stimulates the expression of loricrin, γH2AX, and G6pDH protein expression in skin explants, whereas co-treatment with rose extract prevents these stress manifestations. (**B**) The expression levels of mRNA encoding for OR2AG2 and OR10A6 significantly decrease with epinephrine treatment (**OR2AG2, *OR11H4), while results for OR10A6 did not reach statistical significance. Co-treatment with rose extract significantly inhibits the stress-induced decrease in mRNA expression levels of OR2AG2 and OR11H4, while results for OR10A6 did not reach statistical significance. (**C**) The protein expression of OR11H4 remains unchanged by epinephrine-induced stress, whereas expression of OR2AG2 and OR10A6 (** OR2AG2, ** OR10A6) is significantly reduced. Co-treatment with rose extract significantly inhibits the stress-induced decrease expression levels of OR2AG2. Data are presented as mean ±SEM values. Statistical analysis was performed using ANOVA (Kruskall–Wallis, * *p* ≤ 0.05, ** *p* ≤ 0.01, and *** *p* ≤ 0.001) to compare each group.

To study the potential involvement of ORs in skin response to chronic stress, an ex vivo epinephrine-induced stress model was designed. Three different markers, known to increase under stress conditions, were measured to estimate the stress response of skin (Figure 3A). G6PDH was used as a marker for cell metabolism [26,27], loricrin for cell differentiation [28,29], and γH2AX for DNA damage [30,31].

**Figure 4:**
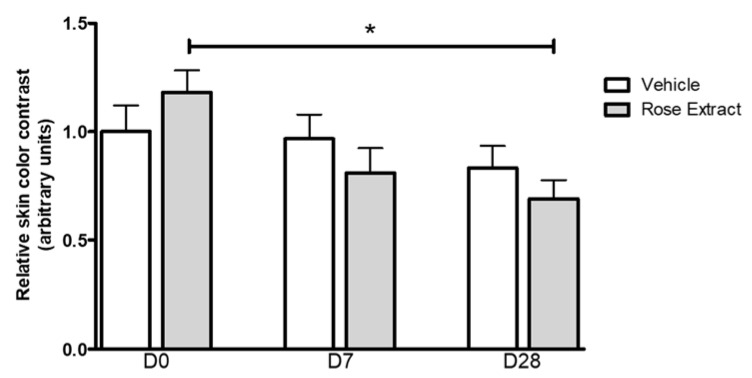
Efficacy of rose extract (5 × 10^−3^%) treatment for under eye dark circles. Skin color contrast evolution between normal skin and dark circles. Data are presented as mean ±SEM values, (*n* = 8). Statistical analysis was performed using ANOVA (* *p* ≤ 0.05).

The following observations were made: first, epinephrine treatment (56 nM) triggers a stress reaction in skin explants, as shown by a significant increase in the expression levels of stress makers. The fold change values are: 2.3 ± 1.17 (*p < 0.05), 1.7 ± 0.47 (* *p* < 0.05) and 1.26 ± 0.19 (* *p* < 0.05) for loricrin, γH2AX, and G6PDH, respectively (Figure 4A). Second, epinephrine-induced stress prompts a downregulation of mRNA levels of OR10A6, OR2AG2, and OR11H4 with fold change values of 0.45 ± 0.2, 0.51 ± 0.3 (** *p* < 0.01), and 0.52 ± 0.2 (* *p* < 0.05), respectively (Figure 3B). In addition, at the protein level, a significant decrease in the expression of OR10A6 and OR2AG2 is also measured, with relative intensity staining of 0.77 ± 0.34 (** *p* < 0.01) and 0.81 ± 0.15 (** *p* < 0.01) for OR10A6 and OR2AG2, respectively. No change in OR11H4 expression is observed (Figure 3C).

Assuming that OR expression is impaired by epinephrine-induced stress, rose extract (5 × 10^−3^%) was used as an OR agonist to see if it could modulate the skin stress response. Results showed that co-treatment with epinephrine and rose extract inhibits the upregulation of G6PDH, loricrin, and γH2AX expression that comes with epinephrine treatment alone (Figure 3A), with fold change values of 0.85 ± 0.19 (** *p* < 0.01), 0.88 ± 0.7 (** *p* < 0.01), and 0.62 ± 0.31 (** *p* < 0.01), respectively. When looking at OR mRNA expression, we saw that adding rose extract (OR agonist) with epinephrine inhibits the decrease normally observed with the stressor (Figure 3B), with fold change values of 0.94 ± 0.56, 0.93 ± 0.26 (* *p* < 0.05) and 2.44 ± 1.84 (** *p* < 0.01) for OR10A6, OR2AG2, and OR11H4, respectively. At the protein level, only OR2AG2 expression (** *p* < 0.01) is increased by the co-treatment, compared to epinephrine treatment alone, reestablishing the level seen in untreated skin (Figure 3C).

Finally, treatment with rose extract alone for 9 days results in an increased expression of mRNAs encoding for OR10A6, OR2AG2, and OR11H4, with fold change values of 2.32 ± 1.0 (*** *p* < 0.001), 3.19 ± 0.72 (*** *p* < 0.001), and 4.09 ± 3.79, respectively (Figure 3B). Surprisingly, no effect could be observed at the protein level (Figure 3C).

### 2.5. In Vivo Effects of Rose Extract Showing a Reduction of Under-Eye Dark Circle Appearance

Rose extract was tested in vivo to document potential cosmetic benefits. A split-face study, on a panel of women volunteers with under-eye bags and dark circles and a stressful lifestyle, was performed. Results obtained with the Bio blue-light 3D scanning technology and images analysis (see Supplementary Materials Figure S3) show that twice daily application of a cream containing rose extract (5 × 10^−3^%) for 28 days significantly (* *p* < 0.01) improves the under eye area and reduces the appearance of dark circles (Figure 4). The fold change values are 0.89 ± 0.36 at D7 and 0.77 ± 0.29 at D28 for rose extract treatment, compared to 0.97 ± 0.32 at D7 and 0.83 ± 0.29 at D28 for treatment with the vehicle alone.

## 3. Discussion

Since the 1990′s, the expression of human ORs has been found in various human normal and diseased tissues [10,11,12,13,14,15,16]. Previous studies have reported the existence and the role of such receptors in keratinocytes [16] and melanocytes [18,19]. Here, we investigated the presence of new skin ORs and the discovery of agonists to address the question of a potential involvement of these receptors in the handling of chronic skin stress.

The current study showed that OR10A6, OR2AG2, and OR11H4 RNA transcripts and proteins are expressed both in vitro in keratinocytes and ex vivo in skin biopsies. These findings are not surprising since other ORs have already been described in skin cells, such as OR2AT4, OR6M1, OR5V1, OR11A1, OR6V1 [16], OR2A4/7, and OR51B5 in keratinocytes [17], or OR2A4/7 and OR51E2 in melanocytes [18,19]. Interestingly, we noticed that the expression patterns of the three ORs differ between skin explants and epidermis. Specific distribution of various ORs was also previously reported in the human eye, where OR10AD1, OR6B3, and OR5P3 can be found in different retina cell types [32]. Similarly, in human spermatozoa, different OR proteins are localized at distinct specialized cellular compartments such as the equatorial segment, the midpiece, and the tail [33]. Cell type specific expression of ORs in various tissues points to a unique role for each receptor.

Even if 3-phenyl propyl propionate and skatole have been identified as OR10A6 and OR11H4 agonists respectively [34,35], no ligand for OR2AG2 has yet been described in the literature to our knowledge. This is a bit surprising given its high level of homology (87%) with OR2AG1 for which ligands such as amyl butyrate, pentyl butyrate, and pyridine derivatives are known [36]. The present study deorphanized OR2AG2 through identification of nine natural agonists, among them phenylethyl alcohol, an agonist for all three ORs, with micromolar range of activity. The latter is a major component of our rose extract. Rose flower is widely used in aromatherapy to bring emotional well-being or reduce nervous tension and stress-related problems, as supported by clinical studies [24,37]. A role in reinforcing the skin barrier function has also been clearly demonstrated [38]. Although the benefits of rose oil are known to be mediated through the olfactory system [39], OR involvement is rarely mentioned. However, the activation of an OR (hOR1A2) with molecules present in a rose oil was already investigated [40] and the authors showed that citronellol and geraniol activate this receptor. Here, for the first time, we confirm that a rose extract activates skin ORs. A previous report [16] showed that activation of OR2AT4 by Sandalore^®^ in keratinocytes triggers an intracellular transduction pathway leading to an increase in cAMP levels. cAMP is described as the canonical downstream messenger released by OR activation during olfaction [3]. Rose extract also triggers a dose-dependent increase in cAMP levels in keratinocytes expressing OR10A6, OR11H4, and OR2AG2. These results strongly suggest that skin ORs could mediate the effect of volatile odorants from the environment, beyond the nose. More specifically, OR10A6, OR11H4, and OR2AG2 expressed in skin cells may help to transduce the physiological appeasing effect of rose extract compounds.

Tabling on the documented positive effect of rose extract on human well-being and the activity found towards ORs, we decided to study its effect on epinephrine-induced stress in skin. To this end, we first measured and analyzed the consequences of such a stress in skin explants. After nine days of epinephrine-induced stress, skin cells develop several mechanisms of defense. As expected, all stress biomarkers were overexpressed. The negative consequences of excessive metabolic activation, dysregulated epidermal differentiation, and DNA damage in the presence of stress can be successfully prevented after the application of the OR agonist. Very few studies have linked OR stimulation with the anti-stress benefits of essential oil inhalation. However, findings by Saito et al. [41] support that the known anti-fatigue effects of a certain mix of odorants involves activation of six olfactory receptors (OR1A1, OR2J3, OR2W1, OR5K1, OR5P3, and OR10A6). Fatigue is a prevalent condition often related to excessive workload and psychological stress. This encouraged us to explore the role of keratinocyte ORs in relation to stress. Interestingly, ex vivo data demonstrated that stress induces a significant down-regulation of the expression of skin OR10A6 and OR2AG2, both at the mRNA and protein levels. Such an effect was already observed for OR2AG2 transcripts which was significantly lower in the lungs of asthmatics compared to normal subjects [42]. Therefore, as seen for the brain [43,44], the skin olfactory system might be affected under stress conditions. When rose extract was present during epinephrine-induced stress, no downregulation was observed in the mRNA levels of skin ORs, while, at the protein level, only OR2AG2 expression followed this trend. Many complicated and varied post-transcriptional mechanisms involved in turning mRNA into protein can explain this phenomenon. Protein half-life, rate of protein synthesis, experimental condition, or protein damages by ROS may contribute to this result [45]. Altogether, these results reinforce the hypothesis that ORs could detect odor information, both in the skin and brain, to engage physiological functions in response to stress. The fact that OR2AG2 seems more affected by stress and rose extract might be the consequence of its localization at the suprabasal layer, which is more susceptible to external stress factors. Moreover, this raises the question of how epinephrine affects ORs and in return how OR activation inhibits epinephrine effects. The fact that ORs might interact with the β2 adrenergic receptor to form a heterodimer, as mentioned by Hague [46] and described for another chemosensory receptor (TAS2R14) by Kim [47], could provide an initial working hypothesis. These authors mentioned that physical interaction and cross-talk between both receptors results in an alteration of receptors conformation, leading to loss of function and responsiveness. Here, activation of ORs, probably OR2AG2, by rose extract may modify β2AR conformation in a way that prevents its interaction with epinephrine and therefore avoids the stress response.

Interestingly, ex vivo results were confirmed with a clinical study showing that rose extract efficiently fights stress-induced skin fatigue. A decrease in the appearance of under-eye dark circles, compared to a placebo, can be observed after both 7 days (−7.2%) and 28 days (−6.5%), in a panel of women volunteers with fine lines on the upper cheeks and a stressful lifestyle. If rose oil was already known to induce relaxing effects following transdermal adsorption [24] and to limit chronic stress-induced disruption of the skin barrier after inhalation [38], the current study adds new scientific data on the benefits of using a specific rose extract in topical application.

## 4. Materials and Methods

### 4.1. Rose Extract and Chemicals

The rose extract under study is a natural extract from *Rosa damascena* mill. flower, containing rose olfactive compounds (hydrophilic and lipophilic). The extract was obtained through 3 successive steps: (1) hydro-distillation of the petals that provides a rose water and rose essential oil, (2) distillation of the rose water that provides more rose essential oil, and (3) purification of the rose water fraction remaining to generate a concentrated rose extract. The 3 fractions thus obtained are recombined in their original proportions, to produce the rose extract whose topical anti-stress activity is documented in this paper. Methyl eugenol, a molecule with a safety issue, is removed in the making process. Rose extract was tested at a concentration of 5 × 10^−3^% in ex vivo and in vivo studies. This low concentration had been chosen based on the first in vitro trials (data not shown). It represents a concentration ten to hundred times more concentrated than the active in vitro concentration. Moreover, as this essential oil is very concentrated and very expensive due to its innovative process, it is not necessary to use a higher concentration on the skin.

Lyral, Peonile, and Cyclemone A were purchased from Pell Wall (Market Drayton, UK), alpha-Damascone from abcr GmbH (Karlsruhe, Germany), and Phenylethyl alcohol from Dr-Straetmans. Sigma Aldrich (St. Louis, MO, USA) was the source for all other odorants.

### 4.2. Rose Extract Analysis

Qualitative and quantitative analysis of rose extract was realized by Gas Chromatography analysis. Briefly, the GC/MS analysis were carried out with an Agilent 6890 Series Gas Chromatograph (Santa Clara, CA, USA) coupled to an Agilent 5973 Mass Selective Detector (MSD, Agilent, Santa Clara, CA, USA), equipped with an automatic liquid sampler and a HP-1MS capillary column (100% dimethylpolysiloxane, 60 m × 0.15 mm i.d., film thickness 0.25 µm; Agilent Technologies, Santa Clara, CA, USA). The oven temperature was held at 60 °C for 10 min, then programmed from 60 to 300 °C at 2 °C/min, and finally 300°C for 10 min (run time 140 min). Ion-source temperature was 230°; transfer-line temperature 260°; ionization energy 70 eV; He (1.0 mL/min) as carrier gas; injector temperature 250 °C with an injection in split mode (1:50). The EI-MS spectral were acquired in the m/z range 40–400 amu.

GC/FID analysis was carried out with an Agilent 6890 Apparatus (Agilent Technologies, Santa Clara, CA, USA) equipped with a flame-ionization detector (FID), an automatic liquid sampler and the same column as described above. The oven temperature was held at 60 °C for 10 min, then programmed from 60 to 300 °C at 2 °C/min, and finally 300 °C for 10 min (run time 140 min). The injector and detector temperature were 250 °C and 300 °C, respectively; H_2_ (1.0 mL/min) as carrier gas; injection in split mode (1:50); injected volume 0.1 mL. The O_2_, H_2,_ and makeup gas flows of the FID were 350, 35, and 20 mL/min., respectively. 

The identification of rose extract components was carried out based on their mass spectra compared to the internal library of compounds.

### 4.3. Keratinocytes Cell Culture and Skin Explants

Skin organ cultures were prepared from tissue samples derived from abdominal or breast tissues of healthy women (30–40 years old) undergoing routine therapeutic procedures, with the donor’s consent. Samples were collected by ALPHENYX (Marseille, France). ALPHENYX possesses the required accreditations from the French Ministry of High Education and Research, in addition to the Committee of Persons Protection under the authorization numbers AC-2014-2141 and DC-2014-2147.

Microdissected human skin was cultured at 37 °C under 5% CO_2_, in DMEM (Dutscher, Brumath, France, #L0060-500) plus 10% FBS (Sigma Aldrich, #F7524), 1% Penicillin/Streptomycin (Sigma Aldrich, St. Louis, MO, USA, #P0781-100), and 1% of minimum medium non-essential amino acids 10X (ThermoFisher, Waltham, MA, USA), #11140-035).

NHEKs cells were isolated from human skin epidermis and cultured in KGM2 Medium (Promocell, Heidelberg, Germany), #C-20011) with 1% Penicillin/Streptomycin (Sigma Aldrich, #P0781-100) at 37 °C under 5% CO_2_ and 95% relative humidity. The detailed protocols are described in the supplementary materials.

### 4.4. Quantitative RT-PCR

For NHEKs, total RNA was isolated and purified using RNeasy Mini Kit (Qiagen, Hilden, Germany, #74104) following the manufacturer’s protocol. For RNA extraction from skin sections, Trizol (ThermoFisher, Waltham, MA, USA, #15596026) was used following the manufacturer’s protocol. Purity was determined with commercial Agilent kit RNA 6000 Nano Kit (Agilent, Santa Clara, CA, USA, #5067-1511) using Agilent 2100 Bioanalyzer according to the manufacturer’s recommendations and the concentration was quantified with a Nanodrop 2000c (ThermoFisher, Waltham, MA, USA). Real-time quantitative polymerase chain reaction (qRT-PCR) was run in triplicate using TaqMan™ Fast Advanced Master Mix (ThermoFisher, Waltham, MA, USA, #4444557) and gene Expression Assay transcripts (ThermoFisher, Waltham, MA, USA) on the AriaMx Real-time PCR System (Agilent, Santa Clara, CA, USA) according to the manufacturer’s recommendations. Real-time quantification plots and Ct values were collected and stored in AriaMx software (version 2.1). The number of transcripts was normalized to that of the housekeeping gene using ΔΔCT method and EXCEL software. All statistical analysis were performed using GraphPad Prism 8 (GraphPad Software version 5.0, San Diego, CA, USA). The detailed protocols were described in the supplementary materials.

### 4.5. Immunocytochemistry (ICC) and Immunohistochemistry (IHC)

For immunocytochemistry staining, anti-OR10A6 (Abcam, Cambridge, UK, #ab129837) or anti-OR11H4 (ThermoFisher, #PA5-562685) or anti-OR2AG2 (ThermoFisher, Waltham, MA, USA, #PA5-538166) primary antibodies were used, all diluted at 1:100. The specificity of the ORs antibodies was not tested on a positive control since a section of the nasal olfactory epithelium was not available. Indeed, the ORs are expressed in olfactory epithelium which constitute only 2 cm² of the nasal cavity and protect the central nervous system, making the biopsy on healthy human very difficult. Revelation were achieved with AlexaFluor 546 (ThermoFisher, Waltham, MA, USA, #A11035) goat anti-rabbit IgG secondary antibody, diluted in 1:800. Moreover, cell nuclei were stained with DAPI (2-(4-Amidinophenyl)-6-indolecarbamidine dihydrochloride, 4′,6-Diamidino-2-phenylindole dihydrochloride) mounting medium (Santa Cruz, Dallas, TX, USA, UltraCruz Mounting Medium, # sc-24941). Negative controls were performed by omitting the primary antibody. Cells were observed using a Zeiss microscope and images were analyzed with ImageJ 2.0 software.

For immunohistochemical staining on human skin, 5-μm-thick sections were made. The same primary antibodies were used (anti-OR10A6 (1:200; Abcam, #ab129837), anti-OR11H4 (1:200; ThermoFisher, #PA5-562685), anti-OR2AG2 (1:100; ThermoFisher, #PA5-538166)). Revelation was achieved with AlexaFluor 488 (green labelling, Abcam #ab150077) or AlexaFluor 546 (ThermoFisher, Waltham, MA, USA, #A11035) goat anti-rabbit IgG secondary antibody, diluted in 1:800. Image processing was performed using ImageJ 2.0 software [48]. For primary G6PDH (1:100 dilution; Sigma Aldrich, St. Louis, MO, USA, #HPA000247), loricrine (1:1600 dilution; Covance, Princeton, NJ, USA, #PRB-145P), γH2AX (1:800; Abcam, Cambridge, UK, #ab26350) antibodies staining, tissue cryosections were fixed on paraffin sections and immunostaining was performed using an automated slide processing system (Autostainer Dako, Agilent, Santa Clara, CA, USA). Vectastain Kit Vector amplifier system avidin/biotin was used, and revealed by VIP, a violet substrate of peroxidase (Vector, Burlingame, CA, USA, #SK-4600). Microscopical observations were realized using an Olympus BX43 microscope. Photographs were digitized with a numeric DP72 Olympus camera and CellD storing software (Waltham, MA, USA). The detailed protocols were described in the supplementary materials.

### 4.6. CRE-Luciferase Assay

Measurements of OR activation are based on the CRE-Luciferase gene reporter assay. HEK293T-RTP1S/RTP2 cells were used for all experiments and were co-transfected with vectors encoding individual ORs and the cAMP reporter gene CRE-Luc vector (pGL4.29 [luc2P/CRE/Hygro]) (Promega, Madison, WI, USA) using TransIT-LT1 Transfection reagent (Mirus Bio, Madison, WI, USA, #MIR2300). Luminescence emission was measured using either a Spectra Max M5 reader (Molecular Devices, Sunnyvale, CA, USA) or a FLUOstar Optima reader (BMG LABTECH GmbH, Offenburg, Germany). The hits, selected according to hit-level (HL) values, were then tested in dose-response experiments using 10 different concentrations from 10^−3.5^ M to 10^−8^ M. Rose extract was tested in the range of 3.2 × 10^−3^% to 1 × 10^−7^%. The concentration response curves were analyzed with Excel or GraphPad Prism 8.3.0 softwares (San Diego, CA, USA). The detailed protocols were described in the supplementary materials.

### 4.7. cAMP-Glo^TM^ Assay

To monitor cAMP production in stimulated NHEKs, the cAMP-Glo^TM^ Assay (Promega, Madison, WI, USA), #V1501) was used according to manufacturer’s instructions. The detailed protocols were described in the supplementary materials.

### 4.8. Chemical Stimulation of Human Microdissected Skin

The study was performed by BIO-EC Laboratory in accordance with the Declaration of Helsinki after the patients had given informed consent to use their skin samples. BIO-EC Laboratory possesses an authorization from the Bioethics group of the General Director Services of the French Research and Innovation Ministry (registered under n°DC-2008-542) to use human skin from surgical waste since 5th May 2010. 

Human skin explants were recovered from the abdominal tissue of a 39 year-old Caucasian woman and kept in a culture medium at 37 °C, under 5% CO_2_. On D0, D2, D5, D7, and D8, explants were stressed by adding 56 nM of epinephrine to the culture medium. Such high concentration of epinephrine has been reported in the plasma of stressed patients [49]. Rose extract (5 × 10^−3^%), diluted in distillated water, was simultaneously added (or not) and skin samples were stored at 4 °C for the duration of the study. Each condition was evaluated on 3 different explants and compared to control (untreated explant). On D0 and D9, explants were collected for evaluation. Tissues were then fixed and embedded in paraffin.

### 4.9. Clinical Evaluation of Rose Extract on Stress-Induced Skin Fatigue

The randomized, double-blinded, split-face study involved a panel of 8 young women aged 25 to 45 years (mean age 32.6 years), presenting under-eye bags and dark circles, and having a stressful lifestyle. Twice a day for 28 days, volunteers applied a cream containing the test product (5 × 10^−3^%) around one eye and a placebo around the other, as instructed. The clinical experiment was performed using a non-invasive skin bioengineering technique. Standardized digital photographs of the treated zones were taken using Bio blue-light 3D scanning technology, at D0, D7, and D28. The region of interest (ROI) was selected and 2 parameters (total area and contrast between normal skin and dark circles under the eye) were obtained. Changes in skin color contrast at D7 and D28, compared to D0, were assessed by image analysis using a dedicated software. Once the image was processed, a threshold was established and the software was able to recognize the dark area under the eye with different level of pigmentation compared to the normal skin. The total area of the dark zone was highlighted and quantified, together with the difference between the grey level in the background (area out of the threshold) and the grey level in the area of the dark spot. The lower this contrast was, the higher was the color uniformity of the ROI.

## 5. Conclusions

The present study supports the notion that the olfactory system is involved in inducing anti-stress responses in skin and provides a new paradigm for the functionality of skin ORs. The extract of *Rosa damascena* mill. flower contains many olfactive compounds, as revealed with the GC-MS and GC-FID analyses, able to activate three skin olfactory receptors. These results show that the rose extract can efficiently decrease skin stress biomarkers, probably through the OR activation. However, the study presents several limitations that should be considered. First, the number of participants in the clinical study was relatively small. Second, further studies using antagonists, or an OR knock-down model would be useful to clarify the role of specific ORs. Generally, the discovery of specific ORs antagonists will be a decisive factor to understand the role of ectopic ORs. Moreover, future immunoprecipitation experiments would be interesting to confirm the hypothesis of a heterodimerization between OR2AG2 and β2AR. It follows that the use of OR agonists could be considered as an alternative to β2AR antagonists in the fight against skin stress. In addition, the rose extract can be used for cosmetic applications to fight stress-induced skin fatigue and to boost natural skin defenses against external and internal daily stressors.

## Figures and Tables

**Table 1:** Volatile constituents identified in *Rosa damascena* mill. flower extract.

Substance	Rt (min)	Compound	Content (%)
1	7.0	Hexanol	0.12
2	8.5	Heptanal	0.04
3	11.6	α-Pinene	0.15
4	13.5	Sabinene	0.03
5	14.3	Beta pinene	0.04
6	15.5	Myrcene	0.07
7	17.1	Benzyl Alcohol	0.55
8	56.7	Phenylethyl alcohol	56.70
9	24.0	Cis-Rose oxide	0.12
10	25.1	Trans-Rose oxide	0.05
11	28.2	Terpinen-4-ol	0.23
12	29.0	α-Terpineol	0.10
13	32.4	Nerol	4.63
14	32.4	Citronellol	16.70
15	32.9	Phenethyl acetate	0.50
16	33.9	Geraniol	8.90
17	34.1	Geranial	0.25
18	35.1	Citronellyl Formate	0.02
19	36.7	Geranyl Formate	0.01
20	39.4	Eugenol	0.15
21	40.1	Citronellyl Acetate	0.40
22	41.6	Neryl Acetate	0.08
23	41.7	Geranyl Acetate	1.06
24	42.4	β-Bourbonen	0.05
25	42.9	β-Elemen	0.08
26	44.4	β-Caryophyllene	0.25
27	45.7	α-Guaiene	0.23
28	46.3	α-Humulene	0.16
29	47.8	Germacrene D	0.25
30	48.9	(−)-α-Selinene	0.06
31	49.3	α-Bulnesene	0.17
32	50.0	Pentadecane	0.11
33	69.1	Nonadecene	0.80
34	70.7	Nonadecane	3.80

**Table 2:** EC50 values (µM) of various natural odorant activators of OR10A6, OR2AG2, and OR11H4.

Entry	Molecules	EC50 (µM)		
		OR10A6	OR2AG2	OR11H4
1	Citronellol	2.0	13.5	NA
2	Nerol	6.0	51.0	NA
3	Cis-3-Hexenol	NA	98.8	NA
4	Benzyl alcohol	NA	NA	NA
5	Linalool	5.6	56.2	NA
6	Geraniol	3.3	18.0	NA
7	α-Cinnamyl alcohol	1.31	8.43	NA
8	Cyclamen aldehyde	9.62	NA	NA
9	Lyral	25.47	NA	NA
10	α-Damascone	NA	NA	NA
11	α-Ionone	7.48	NA	NA
12	Phenyl ethyl alcohol	114.5	151.7	20.3
13	Phenyl propyl alcohol	49.32	103.04	52.84
14	β-Ionone	NA	NA	NA
15	Lavandulol	NA	NA	NA
16	Benzyl acetone	19.10	5.79	NA
17	Cyclemone A	18.66	NA	NA
18	Nonadecane	119.8	NA	NA
19	Rose extract	1.51 × 10^−4^ *	8.12 × 10^−4^ *	1.65 × 10^−4^ *

NA = no activation of the receptor; *n* = 2 experiments. * For rose extract, EC50 values are expressed in % dilution.

## Supplementary Materials

The Supplementary Materials are available online. Figure S1: Immunohistochemistry of olfactory receptors in human skin explants. Figure S2: Normalized dose-response curves of different olfactory receptors (OR10A6, OR2AG2 and OR11H4) expressed in skin, Figure S3: Standardized digital photographs of the treated zones using Bio blue-light 3D scanning technology.

## Abbreviations

RT-qPCR	Quantitative reverse transcription-polymerase chain reaction
G6PD	Glucose-6-phosphate dehydrogenase
γH2AX	H2A histone family member X
cAMP	Cyclic adenosine monophosphate
DNA	Deoxyribonucleic acid
RNA	Ribonucleic acid
DMEM	Dulbecco’s Modified Eagle Medium
KGM	Keratinocyte Growth Medium
ROS	Reactive Oxygen Species
