# Supplementary material for: A Rose Extract Protects the Skin against Stress Mediators: A Potential Role of Olfactory Receptors
Source: Molecules. 2020 Oct 16;25(20):4743. doi: 10.3390/molecules25204743 (PMC7587601; doi:10.3390/molecules25204743)

Supplementary data

A Rose extract protects the skin against stress mediators: a potential role of olfactory receptors

Romain Duroux [1,*], Anne Mandeau [1], Gaelle Guiraudie-Capraz [2], Yannick Quesnel [3] and Estelle Loing [4]

[1] IFF-Lucas Meyer Cosmetics, Toulouse, France
[2] Aix-Marseille University, CNRS, INP, Marseille, France
[3] ChemCom S.A., Brussels, Belgium
[4] IFF-Lucas Meyer Cosmetics, Quebec, Canada
* Correspondence: romain.duroux@lucasmeyercosmetics.com

Supplemental methods

Keratinocytes cell culture and skin explants

Skin organ cultures were prepared from tissues samples derived from volunteers undergoing routine therapeutic procedures, in collaboration with Alphenyx (Marseille). For this study, skin samples were derived from abdominal or breast tissues of healthy women (30-40 years old). Samples were received 1 day post-surgery and used directly for microdissection or cell extraction. Microdissected human skin was cultured at 37°C under 5% CO2, in DMEM (Dutscher, #L0060-500) plus 10% FBS (Sigma Aldrich, #F7524), 1% Penicillin/Streptomycin (Sigma Aldrich, #P0781-100), and 1% of minimum medium non-essential amino acids 10X (Gibco, #11140-035).

For keratinocyte culture, cells were isolated from human skin epidermis. Briefly, skin samples were cut into small pieces and immersed in Thermolysin (Sigma Aldrich, #T7902-100mg) at 4°C overnight. Epidermis was then carefully detached from dermis before being incubated with Trypsin-EDTA for 15 minutes at 37°C. Trypsin inhibitor was next added, the mixture centrifuged and the supernatant discarded before adding KGM2 Medium (Promocell, #C-20011) with 1% Penicillin/Streptomycin (Sigma Aldrich, #P0781-100). NHEK were then maintained in culture at 37 °C under 5 % CO2 and 95 % relative humidity.

Quantitative RT-PCR

For the test, $2.5.10^5$ cells/well were seeded (in a volume of 1.5mL) into 6 well plates, in KGM2 Medium (Promocell, ref C-20011), at 37 °C under 5 % CO2 and 95 % relative humidity for 3 days. Total RNA was then isolated and purified using RNeasy Mini Kit (Qiagen, #74104)

following the manufacturer's protocol. For RNA extraction from skin sections, Trizol (ThermoFisher, #15596026) was used following the manufacturer's protocol. Purity was determined with commercial Agilent kit RNA 6000 Nano Kit (Agilent, #5067-1511) using Agilent 2100 Bioanalyzer according to the manufacturer's recommendations and the concentration was quantified with a Nanodrop 2000c (ThermoFisher). cDNA was adjusted to 1000 ng to allow further quantitative comparison between samples after qRT-PCR. Controls were performed using the housekeeping gene GAPDH. Real-time quantitative polymerase chain reaction (qRT-PCR) was run in triplicate using TaqMan™ Fast Advanced Master Mix (ThermoFisher, #4444557) and gene Expression Assay transcripts (ThermoFisher, Id: Hs01943152_s1 for OR11H4, Hs00358011_s1 for OR1D2, Hs01943871_g1 for OR2J3, Hs01943048_s1 for OR10A6, Hs01004392_s1 for OR4D1, Hs00852484_g1 for OR2J2, Hs01562935_s1 for OR1A1, Hs02338961_s1 for OR2AG2, Hs03045003_s1 for OR51B5, Hs00258239_s1 for OR51E2, Hs02339277_s1 for OR2AT4, Hs00258414_s1 for OR2A4, Hs00243418_s1 for OR1G1, Hs02338915_s1 for OR10J5, Hs01121978_s1 for OR2W1, Hs01943057_g1 for OR10G4 and Hs02786624_g1 for GAPDH) on the AriaMx Real-time PCR System (Agilent) according to the manufacturer's recommendations. Real-time quantification plots and Ct values were collected and stored in AriaMx software. The number of transcripts was normalized to that of the housekeeping gene using ΔΔCT method and EXCEL software. All statistical analysis were performed using GraphPad Prism 8 (GraphPad Software, San Diego, CA, USA).

Immunocytochemistry (ICC) and Immunohistochemistry (IHC)

For immunocytochemistry staining, 5.10^4 cells/well were seeded (in a volume of 500µL) in a Chamber Slide® System Lab-Tek II® (Dutscher, #055078) in KGM2 Medium (Promocell, #C-20011), and incubated for 48 h at 37°C under 5 % CO2. Fixation was then realized with formaline 10% (Sigma, # HT5011-15mL). Keratinocytes were next incubated overnight at 4°C with specific anti-OR10A6 (#ab129837, Abcam) or anti-OR11H4 (ThermoFisher, #PA5-562685) or anti-OR2AG2 (ThermoFisher, #PA5-538166) primary antibodies, all diluted at 1:100 in PBS 1X with 1% BSA. Revelation were achieved with AlexaFluor 546 (ThermoFisher, #A11035) goat anti-rabbit IgG secondary antibody, diluted in 1:800 in PBS 1X with 1% BSA for 1h at room temperature. Moreover, cell nuclei were stained with DAPI (2-(4-Amidinophenyl)-6-indolecarbamidine dihydrochloride, 4′, 6-Diamidino-2-phenylindole dihydrochloride)

mounting medium (Santa Cruz, UltraCruz Mounting Medium # sc-24941). Negative controls were performed by omitting the primary antibody. Cells were observed using a Zeiss microscope and images were analyzed with ImageJ 2.0 software.

For immunohistochemical staining of human skin, 5-µm-thick sections were made using a Leica RM 2125 Minot-type microtome and mounted on Superfrost® histological glass slides. Immunostaining was performed using an automated slide processing system (Autostainer, Dako). For primary G6PDH (Sigma Aldrich, #HPA000247), loricrine (Covance, #PRB-145P), γH2AX (Abcam, #ab26350) antibodies staining, tissue cryosections were fixed on paraffin sections, pre-incubated in PBS-BSA 0.3%-tween 20 at 0.05% with the corresponding primary antibody, overnight for G6PDH (1:100) or for 1h at room temperature in the case of loricrine (1:1600) and γH2AX (1:800). Vectastain Kit Vector amplifier system avidin/biotin was used, and revealed by VIP, a violet substrate of peroxidase (Vector, ref. SK-4600). Microscopical observations were realized using an Olympus BX43 microscope. Photographs were digitized with a numeric DP72 Olympus camera and CellD storing software. Quantification of the proteins of interest (stained purple) was performed through microscopic observation and image analysis. Results were expressed as a percentage of untreated control explants. The relative change (Δ%) between the two tested conditions was also calculated.

To measure OR expression, 5µm sections and slides were permeabilized in PBS with 0.1% Triton X-100. ORs were then specifically labeled, overnight at 4°C with specific anti-OR10A6 (#ab129837, Abcam, 1:200) or anti-OR11H4 (ThermoFisher, #PA5-562685, 1:200) or anti-OR2AG2 (ThermoFisher, #PA5-538166, 1:100) primary antibody, in PBS 1X with 3% BSA. Revelation were achieved with AlexaFluor 488 (green labelling, abcam #ab150077) or AlexaFluor 546 (ThermoFisher, #A11035) goat anti-rabbit IgG secondary antibody, diluted in 1:800 in PBS 1X with 1% BSA, for 1h at room temperature. Moreover, cell nuclei were stained with DAPI (2-(4-Amidinophenyl)-6-indolecarbamidine dihydrochloride, 4′, 6-Diamidino- 2-phenylindole dihydrochloride) mounting medium (Santa Cruz, UltraCruz Mounting Medium # sc-24941). Four sections by condition were cut and 10 to 15 photographs were taken for each section. Image processing was performed using Fiji software (21). We used DAPI dye (blue channel) in order to identify nuclei in the epidermis region. The green channel was used to quantify the fluorescence resulting from OR labelling in the same region. For epidermis segmentation of each image on the green channel, we used a 2D-Gaussian blur filter (1x1

kernel value) for noise removal. Then, we performed a k-means clustering with a number of clusters k = 5, in order to isolate the epidermis+dermis region from both the background and stratum corneum. The segmentation algorithm partitions the input image pixels into k clusters, based on their intensity values. Since both regions were similar in terms of green intensity, we manually selected the dermis region using a circular brush. Then, we used the measurement tool to calculate the mean intensity value within the corresponding dermis selection. To separate both regions (epidermis and dermis), we created a binary region from the k-means image segmentation, using an automatic threshold value corresponding to the mean intensity of the dermis region. This allowed us to obtain a binary image of the epidermis region only. For quantitative analysis, were used the measurement tool to calculate area statistics in the epidermis region. Using epidermis binary image as an inclusive mask, we then computed the surface of the epidermis region (epidermis area size), as well as the level of total intensity in the denoised A488 image (epidermis total intensity). Mean A488 intensity (epidermis mean intensity) was then computed as a ratio between epidermis total intensity and area size. The results are reported as a percentage of the total fluorescence intensity of control.

CRE-Luciferase assay

Measurements of OR activation are based on the CRE-Luciferase gene reporter assay. HEK293T-RTP1S/RTP2 cells were used for all experiments. Cells were seeded on poly-D-Lysine 96-well white plates (Biocoat, Corning, #356691), then co-transfected with vectors encoding individual ORs and the cAMP reporter gene CRE-Luc vector (pGL4.29 [luc2P/CRE/Hygro]) (Promega) using TransIT-LT1 Transfection reagent (Mirus Bio, #MIR2300). Sixteen hours post transfection, the culture medium was replaced by serum-free medium containing the test compounds. After four hours of incubation under carefully controlled conditions, cells were lysed by addition of 50 µL of Lysis buffer and processed for luminescence measurement. Luminescence emission was measured using either a Spectra Max M5 reader (Molecular Devices, Sunnyvale, CA) or a FLUOstar Optima reader (BMG LABTECH GmbH, Offenburg).

Various natural compounds were tested in a primary screening to identify possible pairs between compounds and skin-expressed ORs. Data from the primary screening were analyzed based on their hit-level (HL). This hit-level was calculated using the following formula: HL= $(Luminescence_{compound}-Luminescence_{buffer})/Luminescence_{buffer}$, where $Luminescence_{buffer}$ and

$Luminescence_{compound}$ represent the experimentally measured levels of luminescence of ORs, after application of the test compound and buffer respectively. The hits, selected according to HL values, were then tested in dose-response experiments using 10 different concentrations from $10^{-3.5}$M to 10^{-8}M. Rose extract was tested in the range of $3.2.10^{-3}$ % to 1.10^{-7} %.

The concentration response curves were analyzed with Excel or GraphPad Prism 8.3.0 softwares. EC_{50}, Top and Bottom values, and curves were derived by fitting Y = bottom + (top – bottom) / (1+10^((LogEC50-X))).

cAMP-Glo™ assay

Normal human epidermal keratinocytes (NHEK) were seeded in 96-well plates at a density of 1.10^4 cells/well. After 24 h, cells were treated for 30 min with increasing concentrations of Rose extract, forskolin (Sigma Aldrich, #F3917, 10 μM), or solvent only, at room temperature. To monitor cAMP production in stimulated cells, the cAMP-GloTM Assay (Promega, #V1501) was used according to manufacturer's instructions.

Supplementary Figures

Figure S1. Immunohistochemistry of olfactory receptors in human skin explants.

The distribution of ORs expression was observed with a Zeiss microscope. Red: ORs, Blue: nucleus. Scale bar: 15 µm

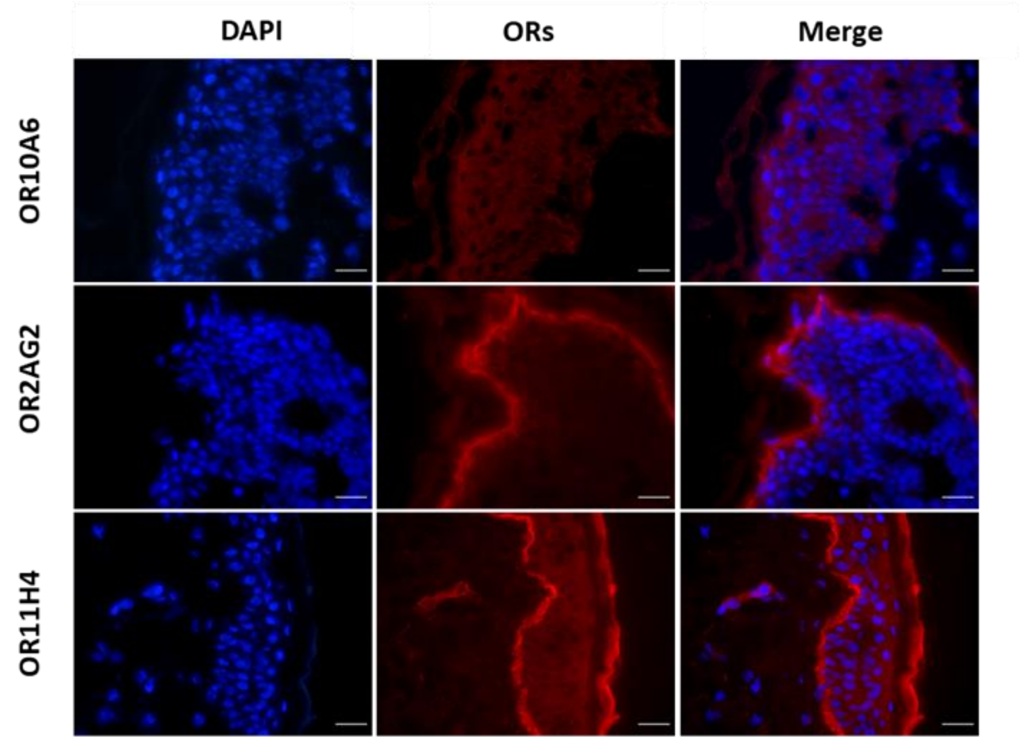

Figure S2. Normalized dose-response curves of different olfactory receptors (OR10A6, OR2AG2 and OR11H4) expressed in skin. Each receptor used for the luciferase assay corresponds to the most common functional allele present in the population. Responses of HEK293T-RTP1S/RTP2 cells transfected with either a plasmid encoding the indicated odorant receptor, or an empty vector, have been normalized such that each receptor has a minimum response of zero and a maximum response of one. Error bars = SD over two replicates. For Rose extract, concentrations are represented in log of % dilution. Abbreviations for the odorants are as follows: RE, Rose Extract; CIT, Citronellol; NER, Nerol; C3HEX, Cis-3-hexen-1-ol; LIN, Linalool; GER, Geraniol; NONA, Nonadecane; PE, Phenylethanol; AMCA, α-Amylcinnamyl alcohol; CA, Cyclamen aldehyde; LYR, Lyral; α-ION, α-Ionone; PEO, Peonile; BA, Benzyl acetone; CYC, Cyclemone A; PPA, Phenyl propyl alcohol.

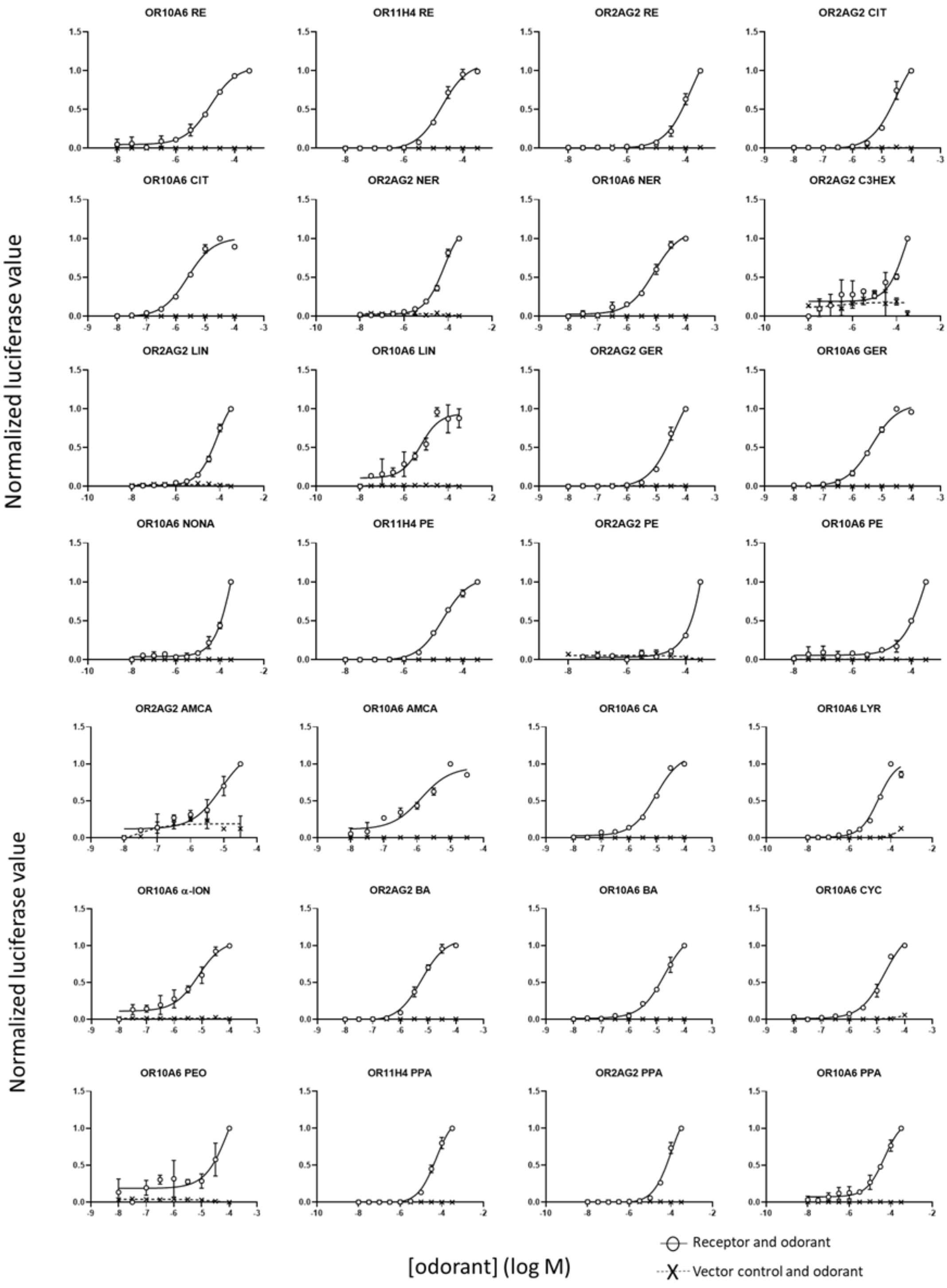
OR10A6 RE
OR11H4 RE
OR2AG2 RE
OR2AG2 CIT
OR10A6 CIT
OR2AG2 NER
OR10A6 NER
OR2AG2 C3HEX
OR2AG2 LIN
OR10A6 LIN
OR2AG2 GER
OR10A6 GER
OR10A6 NONA
OR11H4 PE
OR2AG2 PE
OR10A6 PE
OR2AG2 AMCA
OR10A6 AMCA
OR10A6 CA
OR10A6 LYR
OR10A6 α-ION
OR2AG2 BA
OR10A6 BA
OR10A6 CYC
OR10A6 PEO
OR11H4 PPA
OR2AG2 PPA
OR10A6 PPA
Normalized luciferase value
Normalized luciferase value
[odorant] (log M)
Receptor and odorant
Vector control and odorant

Figure S3. Standardized digital photographs of the treated zones using Bio blue-light 3D scanning technology, at D0 (**A**), D7 (**B**), and D28 (**C**) after rose extract treatment.

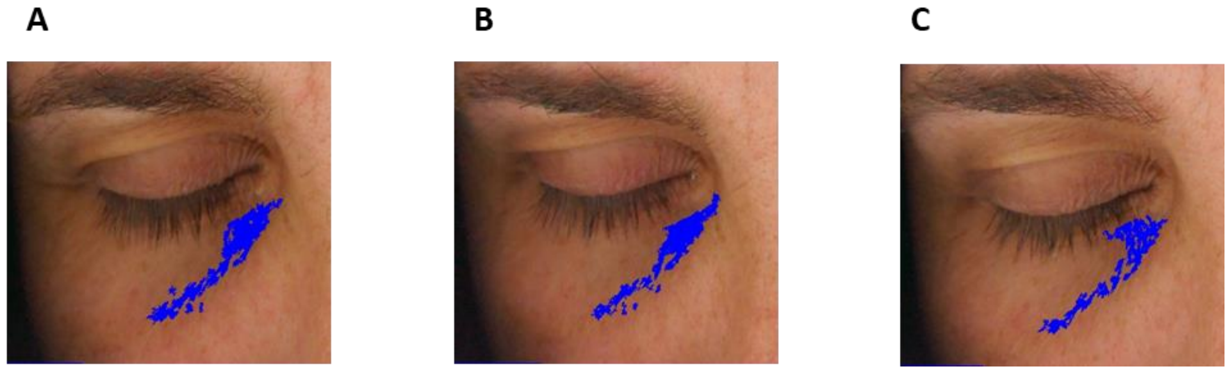

Supplement: Supplementary file 1 [file molecules-25-04743-s001.pdf]
